# Bridging the Accessibility Gap of Cannabinoid Medicine and Arabic Culture

**DOI:** 10.5041/RMMJ.10392

**Published:** 2020-01-30

**Authors:** Dror Robinson, Sivan Ritter, Lilach Zadik-Weiss, Hadile Ounallah-Saad, Nour Abu-Ahmad, Rashid Kashkoosh, Mustafa Yassin, Reuven Or

**Affiliations:** 1 Foot and Ankle Department, Hasharon-Rabin Medical Center, Petah Tikva, Israel; 2 Independent Consultant, Tel Aviv, Israel; 3 Independent Consultant, Amsterdam, The Netherlands; 4 Cannassure Therapeutics Ltd, Ashdod, Israel; 5 Independent Consultant, Nazareth, Israel; 6 Netanya Pain Clinic, Clalit Health Services, Netanya, Israel; 7 Laboratory of Immunotherapy and Bone Marrow Transplantation, Hadassah Hebrew University Medical Center, Jerusalem, Israel

**Keywords:** Arabic, medical cannabis therapy, patient engagement, public health

## Abstract

Arabs are a large minority group in the Israeli society. With the increasing use of medical cannabis throughout Israel due to changing governmental policies, the interactions of the Arab society with medical cannabis becomes of scientific and medical relevance. Recreational cannabis use is considered *haram* (forbidden) in Islam. However, most religious scholars agree that medical cannabis usage might be justified as *zarurat* (emergency and life-saving, therefore allowed) use. Obstacles to medical cannabis use within the Arabic population may relate to language barrier and/or cultural barriers. There are few Arabic-speaking web-based medical-cannabis support groups, and little official information about it is available in the Arabic language. In order for the full benefits of medical cannabis to reach the entire Israeli population, a government-sponsored web-based educational program is necessary in Hebrew and Arabic, both of which are among the nation’s official languages, thereby contributing to the equalization of health resource accessibility.

## BACKGROUND

Patients’ informed consent is a crucial patient right. Informed consent can only be obtained when patients are advised in a clear and understandable language which treatment options are available, as well as their alternatives, risks, prospects, and potential side effects, including those relating to refraining from treatment.^1^ Effects of patient education are well documented in the literature regarding various diseases and studies. Accessibility to information, especially online information gathering, has the potential of fostering greater patient engagement in health maintenance and care.^2^ In contrast to the abundant available information sources regarding medical cannabis therapy (MCT) in various languages, very little available information is easily accessible to the unilingual Arabic-speaking population (in Israel about 1.8 million people [about 24%] are non-Jews and include a number of different, primarily Arabic-speaking groups, each with distinct characteristics^3^). Without resources in a language that patients can read and understand, they cannot achieve the proper health literacy necessary to maintain good health, to make wise health decisions, and to make wise use of health services.^4^

The Israeli Ministry of Health instruction number 7/11 of February 2011^5^ includes, under the topic *standard of care*, cultural competence training and an instruction to promote public health education in minority groups in Israel, also by facilitating collaborations with communities as well as with the local and the religious leadership. The instruction also recommends additional recruitment of professionals from diverse minority background.

New unpublished data (co-author R.K.) compared the differences in populations seeking MCT treatments in two clinics (about 17 km distance apart): one was located in Netanya, a Jewish-majority area, and the other in Qalansawe, an Arab-majority area. During the early days of MCT practice in Israel, out of an average of 20 patients per day visiting the Netanya clinic, ~1–2 patients requested MCT; today that average is ~4–6 patients/day. In contrast, no patients requested MCT in the early days at the Qalansawe clinic, and the current average is ~1–2 patients/day (out of an average 20 patients/day). This information demonstrates the gap in MCT-seeking patients for the Arab population when compared to the Jewish population living in relative proximity to them.

Cannabis (*al-qinnab al-hindi* in Arabic) was introduced into the Middle East mainly from India via Persia; the Greek physicians were familiar with the medicinal properties of the plant and incorporated the plant into their practice. Medical cannabis therapy was practiced by scholars in the medieval Islamic world; however, during the eighteenth and nineteenth centuries the cultivation and use of cannabis were prohibited. Medieval Arab scholars’ documented use of MCT dates from the eighth to the eighteenth centuries, and the *Unani Tibbi* (Arabic-traditional medicine)^6^–^13^ refers to the plant’s diuretic, anti-emetic, anti-epileptic, anti-inflammatory, and pain-killing properties. A seventeenth-century pharmacopeia written by al-Intaqui prescribed cannabis for a number of somatic ailments, but also pointed out its euphoric and lethargic effects.^12^ Despite the scarcity of works dealing with MCT in *Unani Tibbi*, eight *Unani Tibbi* formulas containing cannabinoids were documented by Dwarakanath in 1965.^10^ Currently, most Muslim majority countries enforce a strict prohibition on cannabis use as an axiom of the Islamic prohibition of narcotic or stimulant substances (*haram*); however, a reform of cannabis laws is under consideration in Iran.^13^

The holy Koran does not explicitly forbid cannabis^12^,^13^; therefore, the interpretation of the status of cannabis is very much reliant on the religious scholars’ interpretations. One interpretative category relevant to cannabis use for medical reasons is that of “emergency” (*zarurat*), thus allowing believers to use or to perform generally prohibited substances or acts if these are deemed necessary in situations of emergency, or absolute necessity. This approach is legitimatized based on a Koranic verse (*al-kul maytah*) and on an accepted tradition (*hadith-e raf’*) reiterating that forbidden acts are allowed in times of emergency, if they can be useful and save lives.^13^ One survey of religious scholars’ interpretations regarding cannabis revealed that the majority do not consider cannabis as *haram*, that is to say that it is not totally forbidden; furthermore, the majority of the scholars are of the opinion that if cannabis is used for medical purposes (which must be demonstrated and justified through scientific and medical research), there is no ban on its use (*zarurat*). This contrasts sharply with recreational use, for its intoxicating and inebriating properties. The same study noted that cannabis with high levels of cannabidiol (CBD) (and with no to very low levels of Δ-9-tetrahydrocannabinol [THC]) qualifies as a non-intoxicant substance, and is therefore not prohibited religiously.^13^ Nonetheless, there is a bias against cannabis in Muslim society in accordance with its stereotype (*hashish*, *kif*) for social-recreational use, which is generally forbidden. These combined factors may deter Muslim patients from considering or seeking MCT as a treatment option.

## WHAT TYPE OF PRODUCTS MIGHT BE AVAILABLE?

The predominant intoxicating cannabinoid is THC. Consumption by inhalation of intoxicating cannabinoids is commonly associated with smoking, which is generally associated with recreational prohibited use (*haram*). As stated above, this stereotypical association could deter Muslim and Arab patients from seeking MCT.

Concurrently, it is important to note the increasing number of characterized cannabinoids,^14^,^15^ and the growth in advanced research for future medicinal applications. Despite the intoxicating properties of THC, there are other major cannabinoids that are non-intoxicating and which also offer medical benefits. Cannabidiol, for example, is a much investigated and characterized cannabinoid and has been reported to relieve several medical conditions associated with chronic pain and various inflammatory disorders.^16^ However, the quality of CBD products has been found to be inconsistent; hence the United States Food and Drug Administration (FDA) has tested the chemical content of cannabinoid compounds in some products, and, not surprisingly, they found many products did not contain the levels of CBD claimed in their labeling.^17^

A recent systematic review suggested that CBD may interact with some acute effects of THC. However, the findings were mixed (as CBD did not consistently influence the effects of THC across all studies and outcomes), although CBD was found to reduce the effects of THC in several of the reviewed studies. Moreover, some of the reviewed studies found that CBD reduced anxiety or psychosis-like effects of THC, and blunted some of the impairments of emotion and reward processing.^18^ Therefore, end-products free of THC effects could also potentially qualify as *zarurat* (allowed).

There is an urgent need to develop standardized, consistent, and stable cannabinoid-based products. The FDA has committed to assist in facilitating and preserving incentives for clinical research.^19^ However, to date, they have approved only one CBD product, a prescription drug product to treat two forms of epilepsy. Some CBD products are being marketed with unproven medical claims and are of unknown quality. It is currently illegal to market CBD by adding it to a food or labeling it as a dietary supplement; the FDA has seen only limited data on CBD safety, which points to real risks that must be considered before taking CBD for any reason.^20^

It is therefore a challenge for the evolving medical cannabis industry (MCI) to develop evidence-based safe products (free of pollutants and of contaminants)^17^,^21^–^23^ that have a positive clinically significant effect. Another challenge the MCI faces is the development of a variety of administration routes that do not involve smoking, thereby addressing some of the associated stigma and bias issues.^24^ There are products that are consumed *per os*, as well as dermal patches and other parenteral means of administration available where cannabis has been legalized for recreational as well as medical use. However, it is noteworthy that these products do not always meet the quality standards acceptable by the pharmaceutical industry.^17^ Therefore, in our view the MCI should aim to develop products (oral or parenteral) with standardized content, a characterized manufacturing process, and a defined shelf-life. These products should also be supported by valid clinical studies proving their safety and efficacy.

The growth and survival of MCI relies on developing novel products that address different patient needs. Addressing these needs, including improving appropriate communication, will help both care-givers and patients who are reluctant to seek MCT (due to social or religious banning) to feel more comfortable, by providing an acceptable mode of administration and high-quality information in the Arabic language for the benefit of unilingual Arabic speakers.

## CANNABIS AND THE WEB

Concurrent with the Zeitgeist, patients nowadays form communities, support-groups, and associations organized mainly on social networks. In Israel, such patients’ communities together with their physicians have played a role in the medicalization of cannabis. These communities are active in the Hebrew language, which is one of the official languages in Israel. It is the language spoken by most of the population in Israel. There are many websites on the global Internet; however, the authors of this article did not find equivalences (in content quality, as well as in numbers of websites) when searching popular search engines for the search words “medical cannabis” (الطبي القنب/الهندي القنب) in Arabic language. In terms of public health this warrants both attention and action. Patients who actually seek online information, communal support, and interaction to ease their worries, but only speak Arabic, do not have available resources or social platforms to turn to.

## CONCLUSIONS

Cannabis use in Israel is spreading. However, due to language and cultural barriers, cannabis use by the Arab minority population is limited. In order to encourage better participation in the Israeli healthcare system by Arab-speaking citizens, it is necessary to provide patients with better access to Arab-language web-based health information. It is important to differentiate between its forbidden (by both secular Israeli law and Islamic religious law), recreational use, and its medical use, the latter of which can be justified as emergency use. The government should develop programs for Arab-language information regarding medical cannabis. This will allow diversification of the patient base and equal access by all population sub-groups.

## Abbreviations

CBD: cannabidiol
FDA: United States Food and Drug Administration
MCI: medical cannabis industry
MCT: medical cannabis therapy
MOH: Israeli Ministry of Health
THC: Δ-9-tetrahydrocannabinol.
